# Synergistic Effect of Growth Factor Releasing Polymeric Nanoparticles and Ultrasound Stimulation on Osteogenic Differentiation

**DOI:** 10.3390/pharmaceutics13040457

**Published:** 2021-03-27

**Authors:** Minki Jin, Bo Seok Kim, Sung Ho Seo, Minjeong Kim, Yun Gyeong Kang, Jung-Woog Shin, Kwan Hyung Cho, Meong Cheol Shin, Changhan Yoon, Kyoung Ah Min

**Affiliations:** 1 College of Pharmacy and Inje Institute of Pharmaceutical Sciences and Research, Inje University, 197 Injero, Gimhae 50834, Gyeongnam, Korea; jmk2720@nate.com (M.J.); chokh@inje.ac.kr (K.H.C.); 2 College of Pharmacy and Institute of Drug Research and Development, Chungnam National University, 99 Daehak-ro, Yuseong-gu, Daejeon 34134, Korea; 3 Department of Nanoscience and Engineering, School of Biomedical Engineering, Inje University, 197 Injero, Gimhae 50834, Gyeongnam, Korea; utxtion@me.com (B.S.K.); blussh@onnea.co (S.H.S.); 4 Department of Biomedical Engineering, Inje University, 197 Injero, Gimhae 50834, Gyeongnam, Korea; jjmmjj6827@naver.com (M.K.); 32dbsrud@gmail.com (Y.G.K.); sjw@inje.ac.kr (J.-W.S.); 5 College of Pharmacy and Research Institute of Pharmaceutical Sciences, Gyeongsang National University, 501 Jinju Daero, Jinju 52828, Gyeongnam, Korea; shinmc@gnu.ac.kr

**Keywords:** osteogenic differentiation, mesenchymal stem cells, poly (lactic-co-glycolic acid) nanoparticles, lamin A/C, low-intensity pulsed ultrasound

## Abstract

Mesenchymal stem cells (MSCs) have been extensively used in the tissue regeneration therapy. Ex vivo therapy with well-differentiated osteogenic cells is known as an efficient treatment for musculoskeletal diseases, including rheumatoid diseases. However, along with its high cost, the current therapy has limitations in terms of restoring bone regeneration procedures. An efficient process for the cell differentiation to obtain a large number of functionalized osteogenic cells is necessary. Therefore, it is strongly recommended to develop strategies to produce sufficient numbers of well-differentiated osteogenic cells from the MSCs. In general, differentiation media with growth factors have been used to facilitate cell differentiation. In the present study, the poly (lactic-co-glycolic acid) (PLGA) nanoparticles incorporating the growth factors were included in the media, resulting in releasing growth factors (dexamethasone and β-glycerophosphate) in the media in the controlled manner. Stable growth and early differentiation of osteogenic cells were achieved by the PLGA-based growth factor releasing system. Moreover, low intensity pulsed ultrasound was applied to this system to induce cell differentiation process. The results revealed that, as a biomarker at early stage of osteogenic cell differentiation, Lamin A/C nuclear protein was efficiently expressed in the cells growing in the presence of PLGA-based growth factor reservoirs and ultrasound. In conclusion, our results showed that the ultrasound stimulation combined with polymeric nanoparticles releasing growth factors could potentially induce osteogenic cell differentiation.

## 1. Introduction

Musculoskeletal diseases, such as osteoarthritis, rheumatoid arthritis, or osteoporosis, commonly involve much pain and discomfort recurrence due to imperfect bone restorative procedures [1]. Incompetent bone regeneration could result in bone fractures. Bone regeneration procedure is a reformative process accompanied by inflammation and bone remodeling that involves a number of bone progenitor cells [1]. Autografts or allografts have been used as treatment for bone defects. However, these treatments have several limitations. For instance, autografts could induce post-surgery problems such as infections, chronic pains, or nerve injuries, while allografts could potentially cause immune responses, infections, or other diseases [2]. As an alternative therapy of the conventional treatments, osteoinductive biomaterials substitutes have recently become known as an efficient treatment with less immune rejections or post-surgical complications [3]. Previously, many studies have demonstrated that scaffolds could be devised by containing biomaterials and stem cells as bone progenitors [4,5]. Furthermore, those scaffolds could be constructed by combining bioactive molecules in the nano- or micro-sized particles in the materials to heal bone trauma or defects [6].

Mesenchymal stem cells (MSCs) have been widely studied to develop treatments for various diseases, including tissue regeneration, as these cells can undergo self-renewing process, enabling cell differentiation into osteoblasts, chondrocytes, adipocytes, or other cell types [7,8,9]. In order to differentiate MSCs into specific cell types, various culture conditions were studied using specialized medium compositions or biomatrices [4]. Specifically, in order to use MSCs for bone repairs, growth factors such as bone morphogenetic protein-2 or TGF-β have been studied [6,10]. However, these molecules have some drawbacks in terms of their clinical application. Among these limitations, there are their high costs related with short half-lives, high dosages, or deactivation under in vivo environment. Small functional molecules such as dexamethasone or β-glycerophosphate have been used in the medium for MSC osteogenesis in previous reports [11,12]. These molecules have the advantages of low cost and high physicochemical stability both in vitro and in vivo [13]. Dexamethasone has been used in vitro as an osteogenic factor, while β-glycerophosphate has been known to promote MSC mineralization and differentiation into osteoblasts.

Conventionally, the medium composed of dexamethasone, β-glycerophosphate, supplemented with ascorbic acid has been used for MSC differentiation. These growth factors have been demonstrated to induce the MSC differentiation towards osteoblastic or chondroblastic cells and facilitate those cells to proliferate in vivo [7,14]. However, the culture system with the conventional medium neglects the in vivo microenvironment surrounding the progenic cells that regulates stem cell survival, self-renewal process, and differentiation. The key niche includes the release of growth factors regulated in space and time encountered by stem cells and the mechanical vibration of the nearby tissues [15,16]. The stages of cell differentiation and exposure to growth factors and mechanical force could regulate stem cell function after transplantation in vivo [17]. In the present study, we regulated the niche by using a controlled-release system of growth factors and ultrasound for the cell differentiation at the early stage.

Due to the wide range of application in biological systems and the low cost of devices, ultrasound has been recognized as an attractive tool to complement conventional facilities for clinical or research purposes [18]. For therapy, high intensities (>100 mW/cm^2^) of ultrasound have conventionally been used. However, the low-intensity pulsed ultrasound (LIPUS) with the intensity below 100 mW/cm^2^ could be used for therapeutic purposes within diagnostic level [19,20]. Several previous studies have demonstrated that LIPUS can complement tissue engineering methods for tissue restoration [21,22]. However, the biological effects of LIPUS for the MSC differentiation remain poorly understood.

For the earlier phase of the MSC differentiation into osteoblastic cells, the delivery of growth factors should be well-controlled by the vehicles. Conventionally, for the controlled delivery of various types of drugs, PLGA (Poly(lactic-co-glycolic acid))-based nanoparticles or microparticles have been used [23,24,25]. There is evidence that PLGA could be a useful polymeric tool for bone reformation due to controlled degradability and controlled release properties of drug molecules or proteins [13,26]. Therefore, PLGA-based growth factor delivery could be a safe and efficient way for cell differentiation. In addition to the controlled-release system of growth factors by PLGA, the LIPUS system as a mechanical vibration could be applied to the cell culture for the MSC differentiation.

In the present study, we developed growth factor (G)-encapsulated PLGA nanoparticles and evaluated physicochemical properties of particle systems. We verified the release patterns of growth factors from the PLGA particles in the physiological buffer. Furthermore, we confirmed the effects of combination of low-intensity ultrasound and the G-encapsulated PLGA system for the MSC differentiation by examining the expression levels of Lamin A/C. Since the Lamin A/C is known to become intensively expressed as osteogenic differentiation occurs [27,28], it was selected as a biomarker of early phase of the MSC differentiation into osteoblasts.

## 2. Materials and Methods

### 2.1. Materials

All reagents for experiments were used unmodified and as provided by the manufacturers. Poly (lactic-co-glycolic acid) (PLGA) reagents were obtained from Sigma Aldrich Co. (St. Louis, MO, USA). For particle preparations, various types of PLGA composed of lactide/glycolide monomers with 50/50, 65/35, or 75/25 ratios were used: Resomer^®^ RG 503 H PLGA 50/50 with molecular weight (MW) of 24–38 kDa, PLGA 65/35 with 40–75 kDa (MW), Resomer^®^ RG 756 S 75/25 with 76–115 kDa (MW). Dexamethasone, β-glycerophosphoric acid, and L-ascorbic acid were purchased from Sigma Aldrich Co. (St. Louis, MO, USA). Dichloromethane was obtained from Samchun chemicals (Seoul, Korea), and polyvinyl alcohol (PVA) were purchased from Acros Co. (St. Louis, MO, USA). Acetonitrile, methanol, and water in HPLC grade were obtained from Burdick-Jackson co. (Muskegon, MI, USA) for the LC/MS analysis. Dulbecco’s Modified Eagle medium with low glucose (DMEM-LG), a fetal bovine serum (FBS) and 1% penicillin/streptomycin (P/S) were purchased from Thermo Fisher Scientific (Gibco, Gaithersburg, MD, USA).

### 2.2. Preparation of G-PLGA Nanoparticles

The growth factor-entrapping PLGA (G-PLGA) particles were prepared by an improved double-emulsion (water-in-oil-in-water) solvent evaporation technique [23]. First, 3% PLGA solution was prepared by solubilizing the PLGA powder (150 mg) in 5 mL of dichloromethane. The mixture of growth factors was prepared by mixing 85 μL of dexamethasone (0.0196 mg/mL) and 415 μL of β-glycerophosphate (172 mg/mL) (W1 phase solution). The mixture of growth factors was added into 5 mL of 3% PLGA solution in dichloromethane, which was followed by sonication by the Sonopuls probe with 25% amplitude for 30 s (3 s “on” and 1 s “off”). This W1/O (water 1-in-oil) emulsion was then mixed with 10 mL of 1% PVA solution by the Sonopuls probe with 25% amplitude for 10 min (3 s “on” and 1 s “off”). The W1/O/W2 (water 1-in-oil-in-water 2) emulsion was mixed with 100 mL of 0.3% PVA solution. While 100 mL of 0.3% PVA solution was stirred in the beaker at 1000 rpm, the W/O/W emulsion was added by the syringe via drop-off at the speed of 1 mL/min. After 10 min-stirring, the stirring speed was decreased to 800 rpm, and the emulsion was air-dried overnight to evaporate the remaining dichloromethane. The final G-PLGA emulsion was made into powder by the spray dryer (inlet temperature: 80 °C, outlet temperature: 40–50 °C, pump condition at 15). The final product was stored at 4 °C until further use.

### 2.3. LC/MS Analysis for Quantification of Growth Factors in PLGA Nanoparticles

Calibration curves of β-GP or dexamethasone were prepared with the standard solution of each compound in methanol in the concentration range of 0–1000 ng/mL. LC/MS analysis was performed in a positive mode for β-GP or dexamethasone. The Agilent Poroshell 120 EC-C18 column maintained at 30 °C (column oven temperature) was used for further analysis. The mobile solvent A consisted of water with 0.1% formic acid; the mobile solvent B was prepared as methanol with 0.1% formic acid. For the analysis, mobile solvents were flown at the flow rate of 0.3 mL/min.

The entrapment efficiency was calculated following a previous study [29]. To estimate the entrapment efficiency of G-PLGA particles, 20 mg of total amount of the G-PLGA particle powder in each batch were vortexed with 0.5 mL of methanol for 1 min. Then, the sample was mixed with 2.5 mL of HPLG-graded water by vortexing for 2 min. After centrifugation at 7000 rpm for 5 min, the supernatant was added to the Captiva EMR-Lipid filter cartridge, and the elution was obtained by a vacuum. The solution was filtered via 0.22-μm PVDF syringe filters and injected to the 6460 Agilent QQQ LC/MS for the quantitation of β-glycerophosphate (β-GP) or dexamethasone entrapped in the particles in each batch. Loaded amount of dexamethasone or β-GP in 20 mg of G-PLGA particle powder was approximately 0.1 μg or 9 μg, respectively. The entrapment efficiency was calculated using Equation (1).

(1) $$\mathrm{Entrapment}\;\mathrm{efficiency}\;\left(\%\right)\;=\;\frac{\left(Total\;added\;amount\;of\;G\right)\;-\left(Amount\;of\;free\;non-entrapped\;G\right)}{Total\;added\;amount\;of\;the\;growth\;factor\;\left(G\right)}$$

### 2.4. Physicochemical Characterization of Particles

#### 2.4.1. Measurements of Particle Properties by Dynamic Light Scattering (DLS) and Scanning Electron Microscopy (SEM)

A NanoBrook 90Plus DLS instrument was used to measure particle size and zeta potential values [30]. G-PLGA particles were dispersed in water in the appropriate concentration before the measurement. BI-ZEL electrode set was used for the zeta potential measurement. To evaluate the morphologies of particle formulations, field emission scanning electron microscopy (FE-SEM; S-4700, Hitachi, Tokyo, Japan) was used with the samples on the carbon tape.

#### 2.4.2. Characterization of Particle Wetting Properties by a Microscopy

In order to demonstrate dispersibility of G-PLGA particles in the aqueous solution, particle wetting behaviors were examined using the Zeiss light contrast microscopy (Axio observer Z1, Carl Zeiss MicroImaging GmbH, Jena, Germany). Particle powder was placed on the slide glass, and then a drop of basal medium was added by a pipette. Next, the wetting phenomenon was captured by the microscopy.

#### 2.4.3. Measurements of Particle Properties by Dynamic Scanning Calorimetry (DSC) and X-ray Diffraction (XRD)

Differential scanning calorimeter analysis was performed for particle formulations using a TA Instruments Q20 DSC [31]. After the calibration of instrument was performed using indium, samples in an aluminum pan were analyzed at the heat rate of 10 °C/min under nitrogen gas at 20 mL/min. Furthermore, the particles were analyzed by an X-ray diffractogram (XRD) to get any crystalline characteristics [32]. The XRD instrument, Ultima IV diffractometer (Rigaku, Japan), was set at the voltage at 45 kV, and the current at 30 mA, scanning the diffraction angle of samples between 0° and 90°.

### 2.5. Low-Intensity Ultrasound Setup

**Figure 1:**
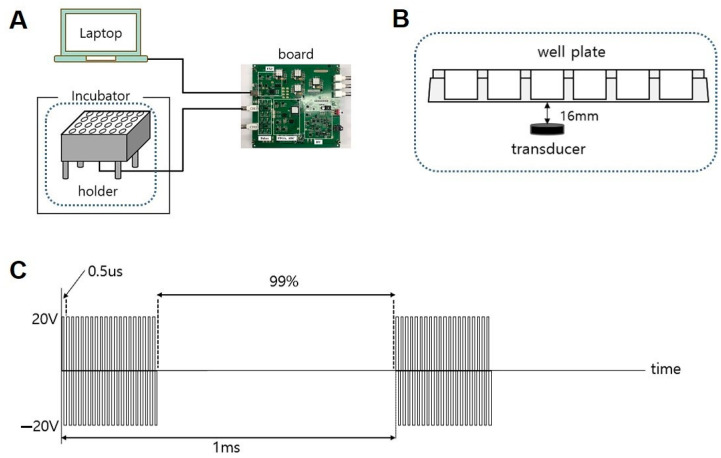
Low-intensity pulsed ultrasound (LIPUS) setup diagram: (**A**) ultrasound setup with the cell culture plate in the incubator; (**B**) enlarged setup of the cell culture well plate over the ultrasound transducer; (**C**) pulse repetition frequency settings in time.

As can be seen in Figure 1, low-intensity pulsed ultrasound (LIPUS) was manufactured in a 24-well plate according to a previously reported protocol [33,34]. The distance between the transducer and the plate was 16 mm and was filled with distilled water in order to deliver good ultrasound (Figure 1A,B). LIPUS was set to pulse repetition frequency (PRF) at 1kHz, frequency at 2 MHz, pulse duration at 1%, transmit voltage at 20 V (Figure 1C). Ultrasound stimulation was applied to the bottom of the culture plate in the CO_2_ incubator twice a day for 30 min and at a time interval of 5 h.

### 2.6. Release Study of Growth Factors from G-PLGA Particles

In order to evaluate release profiles of growth factors from G-NPs, the experimental setup of drug release studies was modified from a previously reported method [29]. G-NP dispersion (20 mg) in 1 mL of PBS buffer (pH 7.4) was loaded in the 24-well culture plate. Then, a Transwell insert with polyester membrane (pore size: 0.4 μm) was placed in the plate with the blank PBS buffer (200 μL) added to the insert. The plate with the insert was incubated in 37 °C incubator with shaking or ultrasound (LIPUS) until Day 10. For the comparison, this experiment was performed with G-NPs without physical agitation (i.e., no shaker or LIPUS). During the experiment, the sample (50 μL) was taken at each time from the insert, and the insert side was refilled with a blank PBS buffer with the same volume of the sample volume taken. All samples were extracted with methanol, filtered by a PVDF syringe filter, and, finally, injected to LC/MS for the quantitation of growth factor concentrations. This experiment was performed for three different batches of G-NPs and finally, the percentage cumulative release of the growth factor was calculated using Equation (2) [35].

(2) $$\mathrm{The}\;\mathrm{cumulative}\;\mathrm{release}\;\left(\%\right)\;=\;\frac{Total\;amount\;of\;growth\;factor\;released\;at\;time}{Initial\;loaded\;amount\;of\;the\;growth\;factor}\times 100$$

### 2.7. Cell Culture

Human mesenchymal stem cells (MSCs) (Lonza, Switzerland) were cultured on a 75-cm^2^ flask at the cell density of 5000 cells/cm^2^ using a basal growth medium (Dulbecco’s Modified Eagle medium with low glucose (DMEM-LG) supplemented with 10% fetal bovine serum (FBS) and 1% penicillin/streptomycin (P/S)) in 37 °C, 5% CO_2_ incubator. When the cell confluency exceeded 80%, the cells were seeded on the chambered glasses at the cell density of 5000 cells/cm^2^ using trypsin/EDTA (Lonza). Finally, passages #4–#5 cells were used for cellular experiments. The media were replaced every 2–3 days.

### 2.8. Examination of Osteogenic Differentiation from Mesenchymal Stem Cells (MSCs)

#### 2.8.1. Application of Different Cell Culture System for Differentiation

Osteogenic differentiation medium (DM) was consisted of basal DMEM-LG medium containing 10% FBS, 1% P/S, 10^−7^ M dexamethasone, 50 μg/mL L-ascorbic acid, and 10 mM β-GP. As another type of culture system for MSC differentiation, 4 mg of G-NPs were added to the basal medium containing 10% FBS, 1% P/S, and 50 μg/mL L-ascorbic acid. Experimental groups were classified into the following three groups: (1) control group (cells cultured in a basal medium containing only 10% FBS, 1% P/S); (2) differentiation medium (DM) group; and (3) G-NP-based medium group (the basal medium containing 10% FBS, 1% P/S, and 50 μg/mL L-ascorbic acid, supplemented with G-NPs). Differentiation of the three groups was examined with or without LIPUS. Cellular examinations were performed on Day 3 or Day 5 of culture under each culture condition.

#### 2.8.2. Immunofluorescence Study

On Day 3 of culture, the cells on chambered glasses were fixed for 10 min with 10% formalin (Sigma, St. Louis, MO, USA) and washed three times with phosphate buffered saline (PBS; Sigma, St. Louis, MO, USA). The cells were permeabilized by 0.1% Triton X-100 for 5 min and washed with PBS three times. To prevent nonspecific binding of the antibody, 1% bovine serum albumin (BSA; MP biomedicals, Singapore) was treated for 30 min at room temperature. Then the cells were treated with the Lamin A/C antibody (Santa Cruz Biotehcnology, Dallas, TX, USA) diluted in the ratio of 1:100 in 1% BSA at room temperature for 1 h. After washing three times with PBS, the secondary antibody was treated at room temperature for 1 h. As a secondary antibody, Alexa Fluor 488 rabbit anti-mouse IgG (invitrogen, USA) diluted in the ratio of 1:500 in 1% BSA was used. After washing with PBS, the cell nuclei were stained for 7 min using Hoechst 33258 (Thermo Fisher, Waltham, MA, USA). Confocal microscopy (Carl zeiss, Jena, Germany) was used to confirm fluorescent signals of Lamin A/C and cell nuclei with ×40 magnification.

#### 2.8.3. Image Analysis

Image J software ver. 1.5 (NIH, Bethesda, MD, USA) was used to analyze each cell in the confocal microscopic image. One cell was selected from a picture and then copied to a 256 × 256 window, resulting in creating a .tif file. The same procedure was used to obtain photographs of all cells. The MATLAB software (ver.2018b, Mathworks, Inc., Natick, MA, USA) was used to numerically express the intensity of Lamin A/C. The intensity value of Lamin A/C of each cell was expressed as the sum of the Lamin A/C intensity divided by the pixel area of Lamin A/C.

### 2.9. Data Analysis

Microsoft Excel 2010 and GraphPad Prism 5.03 (GraphPad Software; LaJolla, CA, USA) were used for data analysis and statistics. Specifically, one-way ANOVA with the significance level of 0.05 was run as a statistical analysis.

## 3. Results and Discussions

### 3.1. Physicochemial Characteristics of PLGA Particle Formulations

After the preparation, growth factor-containing PLGA (G-PLGA) particles were measured for sizes and surface charges by the dynamic light scattering method to evaluate the physical stability as particle formulations [36]. Those values were measured on different days of storage; furthermore, the sizes and charges of particles in spray-dried powder form were compared with those of the particles dispersed in water during the storage period. Table 1 shows hydrodynamic sizes and zeta potential values of G-PLGA particle formulations on Day 1 of particle preparation and Day 30 after the storage at 4 °C. Spray-dried powder forms of G-PLGA particles prepared from PLGA 50/50, 65/35, or 75/25 (PLA (polylactic acid)/PGA (polyglycolic acid) ratio) polymer types showed little difference in sizes or zeta potentials during a month of storage. Moreover, when the spray-dried powder of particles was dispersed in water and then stored in solution at 4 °C, the particle sizes or zeta potential values showed little change for a month of storage. These results suggest that, when stored as powder form or in solution, growth factor-containing PLGA particle formulations maintained physical stability with about 500 nm sizes.

**Figure 2:**
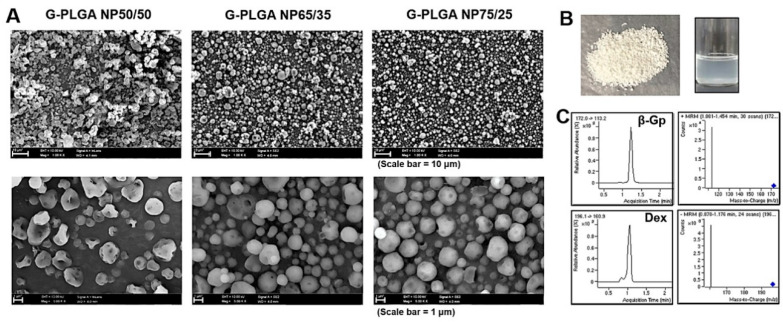
Physicochemical examination of growth factor-containing PLGA nanoparticles (G-PLGA NPs) and growth factor quantitation by LC/MS. (**A**) Particle morphologies (G-NP50/50, G-NP65/35, G-NP75/25) were examined by scanning electron microscopy (SEM). The enlarged SEM images are displayed under each image. (**B**) Powder form or dispersion of the G-NP50/50 in water exhibited as an example. (**C**) Chromatograms of β-GP and Dexamethasone (growth factors) standards by the MRM method of LC/MS. The LC/MS method was used for the quantitation of growth factors entrapped in the PLGA NP.

After particle preparation, G-PLGA nanoparticles (G-NP50/50, G-NP65/35, or G-NP75/25) were examined for the physical shape by scanning electron microscopy (Figure 2A). As can be seen in Figure 2A,B, the spray-dried G-NP50/50, NP65/35, or NP75/25, white-like powder, had nearly spherical shape with small dents. The powder could be well dispersed in water like emulsion.

In order to quantify the concentrations of growth factors (β-glycerophosphate (β-GP) or dexamethasone) entrapped in the PLGA NP50/50, NP65/35, or NP75/25, the LC/MS analysis methods were used with a positive mode. As can be seen in Figure 2C, the MRM (multiple reaction monitoring) method was applied to specifically get the chromatograms of β-GP or dexamethasone. Under the positive mode, the parent ion and product ion m/z ratios of β-GP were 172.0→113.2, and those of dexamethasone were 196.1→160.9. The linearity of the calibration curves of β-GP or dexamethasone was confirmed using the standard solution containing β-GP or dexamethasone, respectively, in the varied concentration range. Calibration curves were used to calculate the concentrations of β-GP or dexamethasone in the G-NP formulations. The encapsulation efficiencies (%) of G-NPs were calculated based on the LC/MS analysis of formulation types. The results showed that the encapsulation efficiencies (%) of dexamethasone in G-NP50/50, NP65/35, or NP75/25 were on average (*n* = 3) 80.4 ± 5.3, 88.8 ± 7.4, or 86.4 ± 3.6, respectively.

**Figure 3:**
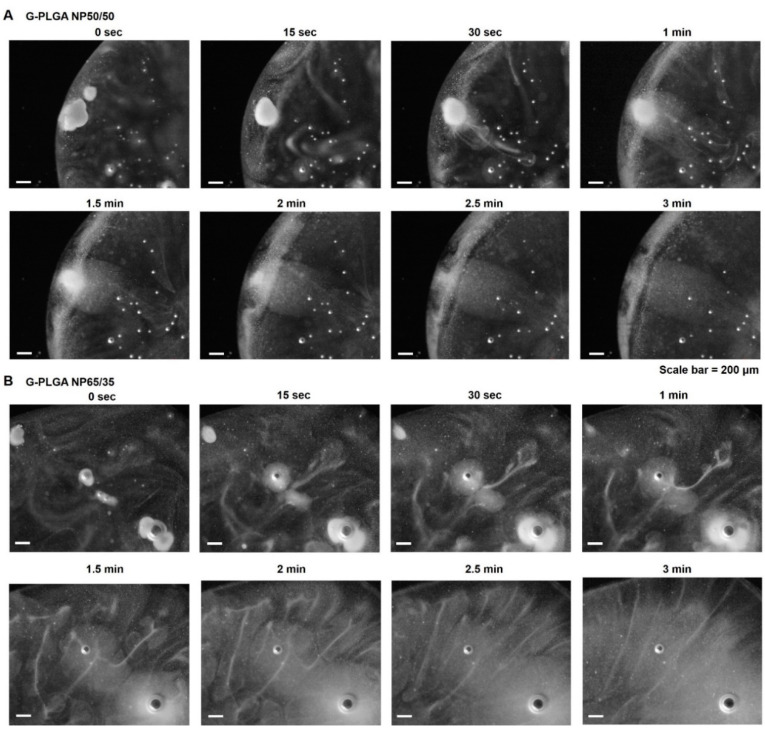
Light contrast microscopic examination of wetting patterns of growth factor-containing PLGA nanoparticles (G-PLGA NPs) in media by time: (**A**) G-NP50/50, (**B**) G-NP65/35.

Since growth factor-containing PLGA (G-PLGA) particles should be used in the culture media as the supplement for the cell differentiation, it is important to evaluate whether the powder forms could be dispersed in solution like emulsion [29,37]. The wetting properties of the powder forms of G-NPs were evaluated by the microscopic examination. In Figure 3A, after a drop of the medium was added to the powder of G-NP on the slide glass, G-NP50/50 started to be wet in 30 s and then, in 3 min, became dispersed in the solution without any particulates. On the other hand, G-NP65/35 showed better wetting behaviors, with efficient wetting in the medium, starting to be wet in 15 s and well-dispersed in 1.5 min (Figure 3B). G-NP75/25 (data not shown) showed similar patterns of wetting in the medium to G-NP65/35, suggesting that PLGA types synthesized with more ratios of PLA to PGA might contribute to better wetting of particle formulations in the aqueous solution.

### 3.2. DSC and XRD Analyses of PLGA Particles

**Figure 4:**
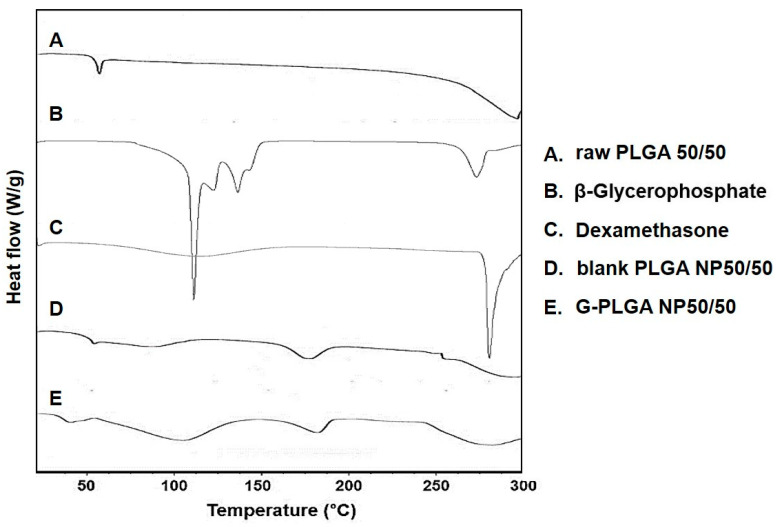
Dynamic scanning calorimetry (DSC) results of (**A**) raw PLGA 50/50 powder, (**B**) β-glycerophosphate, (**C**) dexamethasone, (**D**) blank PLGA NP 50/50, (**E**) growth factor-containing PLGA (50/50) NPs (G-NP50/50).

**Figure 5:**
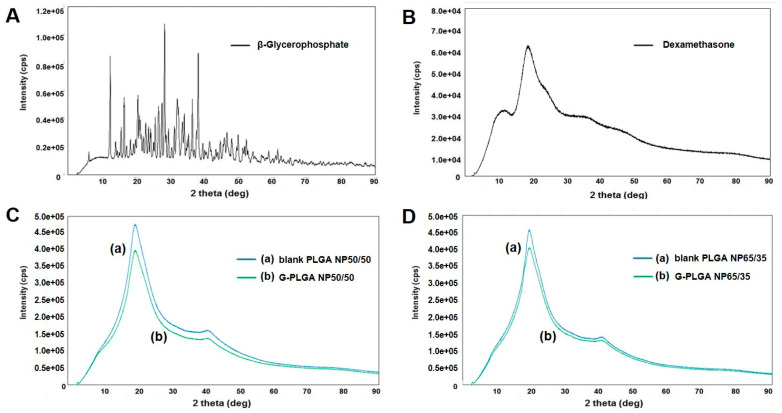
X-ray diffraction (XRD) results: (**A**) β-glycerophosphate, (**B**) dexamethasone, (**C**,**D**) XRD displays of (**a**) blank PLGA NPs vs. (**b**) growth factor-containing PLGA NPs. (**C**) PLGA (50/50) NPs vs. (**D**) PLGA (65/35) NPs.

Generally, DSC and XRD analyses are performed to evaluate whether, after particular formulations are prepared, drug substances entrapped in the particles possessed their physical states as crystals or amorphous forms [38]. In the present study, in order to evaluate the physical states of growth factors (β-GP or dexamethasone) in the G-NPs, DSC and XRD results of G-NPs were compared with raw growth factors, PLGA powder and blank NPs without growth factors. As can be seen in Figure 4, β-GP and dexamethasone showed major endothermic peaks at about 105 °C and 277 °C, respectively. The PLGA NP formulations containing growth factors (G-NPs) showed little difference from NPs without growth factors (blank NPs) in the DSC. As shown in Figure 5, the XRD results of PLGA NP formulations (blank or G-NPs) did not show the characteristic X-ray diffraction properties of growth factors (β-GP or dexamethasone). In general, PLGA is known as an amorphous material, and our observations support this contention. Based on these results, we assumed the preparation process of G-NPs with PLGA can provide particle formulation in which growth factors are well-encapsulated.

### 3.3. Growth Factor Release Profiles of PLGA-Based Formulations

**Figure 6:**
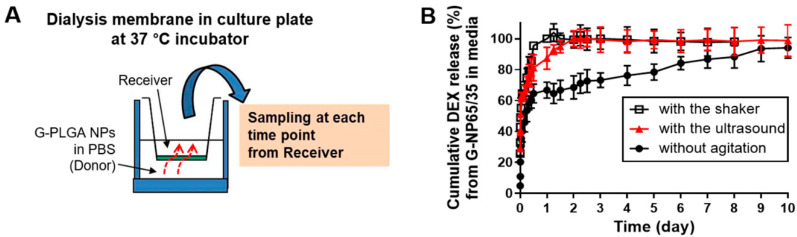
Release profiles of the growth factor (dexamethasone) from G-PLGA NP65/35. Using the setup of (**A**) dialysis membrane insert in the culture plate, the release study of dexamethasone from G-PLGA NPs was performed. (**B**) The release levels (%) of dexamethasone from G-PLGA NPs were measured in the setup with the shaker or ultrasound, or without any physical agitation or force. The cumulative release (%) of dexamethasone from G-PLGA NP65/35 is depicted with the time until Day 10.

Along with the physical evaluation of the growth factor-containing formulations, G-NPs, growth factor release patterns were analyzed. As shown in Figure 6A, the Transwell inserts with porous polyester membrane (pore diameters of 0.4 μm) were located in the culture plate, and G-NP dispersion in PBS buffer (pH 7.4) was added to the donor side. During the incubation at 37 °C, the plates with the inserts were subject to shaking or ultrasound as physical agitation to mimic an in vivo microenvironment that cells could experience before osteogenic differentiation. This setup with physical agitation by a shaker has been widely used for in vitro drug transport studies to make experimental condition similar to in vivo environment [39,40]. In our study, the release patterns of growth factors from G-NPs with the shaker or low-intensity pulsed ultrasound (LIPUS) were compared with the profiles of the growth factors without any physical agitation. Concentrations of the growth factors released from particle dispersion were quantified by the established LC/MS method. The growth factors without particle preparations were released almost 100% in the medium within 5 min after the powder was put in the experimental settings, as the substances dissolved well in the aqueous medium. Figure 6B shows cumulative dexamethasone release (%) from G-NP65/35 formulations until Day 10. Without the agitation or force, the release (%) of dexamethasone reached about 100% at Day 10 and only about 70% at Day 3. This was so because it might take time to erode all the amount of G-NP formulation powder in a well of the culture plate, finally eluting out the growth factors into the medium, under the condition without any agitation or shaking. Without agitation, the concentration of the growth factors in the medium in the receiver side of Transwell inserts might differ from that in the donor side of the well of the culture plate. On the other hand, under the physical agitation (shaking or LIPUS), almost 100% of growth factors were released to the receiver side of inserts by Day 3. Similarly to the shaker, LIPUS could facilitate the release of growth factors from the particle formulations in solution. In the present study, ultrasound was expected to provide the niche for facilitating the MSC differentiation, mimicking in vivo environment and facilitating the release of growth factors into the medium.

### 3.4. Application of G-NPs and Ultrasound for The Osteogenic Differentiation

In the presence of the mesenchymal stem cells (MSCs), growth factor-containing NPs (G-NPs) were investigated in the aspect of the medium supplement for the osteogenic differentiation. Lamin A/C proteins have been regarded as representative biomarkers expressed in nuclear membranes in the early stages of osteogenic process [28,41]. Previous studies tested usefulness of LIPUS, a type of ultrasound, for enhancing bone formation in vitro and in vivo. Cui et al. reported that rabbit MSCs significantly expressed chondrogenic biomarkers as treated with LIPUS at 200 mW/cm^2^ [21]. Hsu et al. demonstrated that proliferation and chondrogenic differentiation of MSCs could be induced by the ultrasound [42].

**Figure 7:**
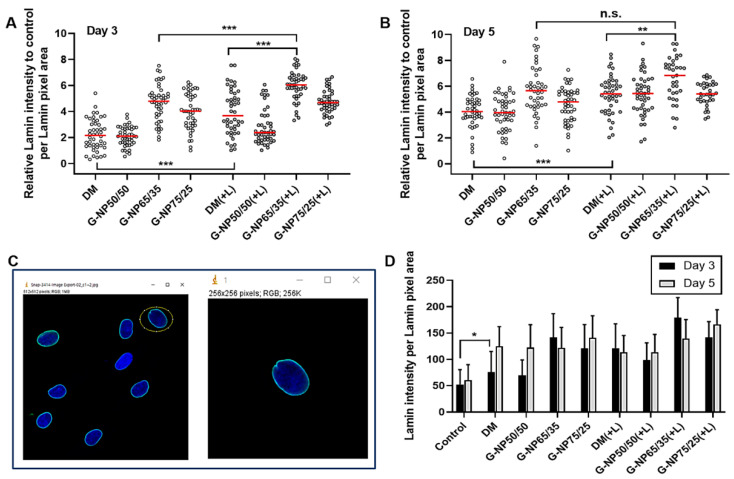
Lamin A/C protein intensity per Lamin pixel area in the treatment group cells (differentiation media (DM), or in the media with G-NP50/50, G-NP65/35, G-NP75/25) during culture period without or with ultrasound, LIPUS (+L). Relative Lamin A/C/intensity per Lamin A/C pixel area was calculated by normalizing the values of Lamin intensity per pixel area of the treatment group by the averaged value of control group cells (Control) on (**A**) Day 3 or (**B**) Day 5 after the culture. (**C**) Lamin A/C intensity value per cell was calculated by MATLAB program based on the confocal microscopic image. (**D**) Lamin A/C intensity per Lamin A/C pixel area values of control or treatment group cells are displayed with averages and standard deviation bars. The presence of LIPUS is indicated as (+L) for the treatment group. The statistically significant differences were compared by one way ANOVA (Tukey’s multiple comparison test as the post hoc test). * *p* < 0.05, ** *p* < 0.01, *** *p* < 0.001, n.s. as not significant.

Herein, we have evaluated the 3-day and 5-day differentiation of MSCs cultured in media containing G-PLGA particles under optimized LIPUS treatment. As a negative control group, control MSCs were cultured in the basal medium without growth factors (β-GP, dexamethasone, or ascorbic acid). As a positive control, MSCs were cultured in the differentiation medium (DM) with growth factors (β-GP, dexamethasone, or ascorbic acid) spiked at the recommended dose for cell cultures. As the treatment group, MSCs were cultivated in the medium containing ascorbic acid, with the G-NPs supplemented in the absence or presence of LIPUS. After cultivation by Day 3 or Day 5, immunofluorescence experiments were performed to stain Lamin A/C proteins in MSCs in each control or treatment group. As shown in Figure 7A,B, relative Lamin A/C intensity per Lamin A/C pixel area was compared in all treatment groups, based on Lamin A/C intensity per Lamin A/C pixel area of control cell group. Those intensity values of Lamin A/C per cell were analyzed by MATLAB program based on the confocal microscopic images (Figure 7C). As compared to the cell differentiation in the differentiation medium (DM) with the growth factors spiked as a general culture method, either ultrasound (+L) or G-NPs (NP65/35 or NP75/25) increased Lamin A/C expression levels of MSCs within Day 3 of culture (Figure 7D). As compared to the cells in the either ultrasound (+L) or G-NP65/35, the combination of LIPUS and G-NP65/35 showed a statistically significant induction of Lamin A/C expression within Day 3. On Day 5, in the presence of LIPUS, the cells with G-NP65/35 in the medium exhibited a higher induction of Lamin A/C expression, as compared to those in differentiation medium (DM) with the growth factors spiked. Overall, ultrasound showed facilitating effects of osteogenic differentiation of MSCs in the early culture period (Figure 7C). In the presence of LIPUS in the culture system (Day 3), MSCs exhibited a higher Lamin A/C intensity per Lamin A/C pixel area in both cases of the general differentiation medium (DM) or the medium supplemented by G-NP65/35.

**Figure 8:**
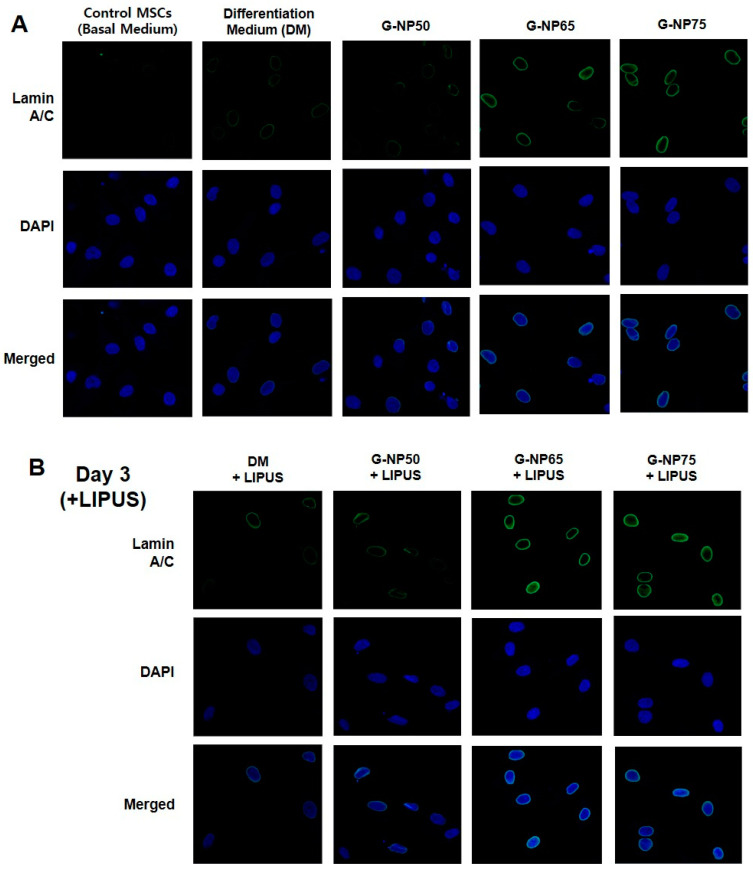
Confocal fluorescent microscopy examination of Lamin A/C expressions and cell nuclei of MSCs. (**A**) Images of Lamin A/C and cell nuclei stains of Control MSCs in the basal medium vs. MSCs in the differentiation medium (DM) with growth factors spiked. The results were compared with MSCs in the medium with growth factor-containing PLGA NP50/50, NP65/35, NP75/25 on Day 3 after the culture. (**B**) Lamin A/C stain and cell nuclei (DAPI) in the presence of the ultrasound (LIPUS) on Day 3 after the culture. The merged images combine Lamin A/C and DAPI results at the same foci.

Confocal microscopic images (Figure 8) demonstrated that Lamin A/C expression levels were induced by G-NPs (G-NP65/35 or G-NP75/25) on Day 3 and were even further increased by the combination treatment with LIPUS. Ultrasound has been used as a safe diagnostic tool in clinics by transferring mechanical energy to biological system [43,44]. Our results demonstrated that the LIPUS with optimal setup could efficiently induce the biological function such as cell differentiation. Unlike other steady mechanical stimulation types as compression or fluid shear stresses, LIPUS can subject the cells to a periodically varied normal stresses and to be stimulated in the regulatory pathways due to acoustic streaming with low intensity ranges. The periodical and non-thermal stimuli by LIPUS has been known to provide the least cytotoxicity issues as well as result in changes in matrix or metabolic function of tissues in vitro and in vivo [45,46,47]. Still, the exact mechanism of positive effects of LIPUS on cells is not completely known. Several studies found that, by LIPUS application, mechanosensitive ion channels can be activated to trigger calcium entry into cells, modulating cytoskeletal structures [48,49]. Aliabouzar et al. demonstrated that synthesis of glycosaminoglycan and collagen in the MSCs increased by LIPUS application, resulting in stable differentiation to cartilage tissues [43]. These studies suggest that LIPUS stimulation could be useful for MSC differentiation.

## 4. Conclusions

In the present study, we developed and evaluated osteogenic factors (dexamethasone and β-glycerophosphate)-encapsulating PLGA particles (G-PLGA NPs) for the efficient culture of mesenchymal stem cells (MSCs) to be used for bone regeneration purposes. In addition, the cell morphologies and osteogenic marker expression were evaluated when a low-intensity pulsed ultrasound (LIPUS) was applied to the cells growing in the conventional culture medium or the medium containing G-PLGA NPs. In the cells cultured in the medium with G-PLGA, the presence of LIPUS enabled a more intensive expression of Lamin A/C protein in the cells in the early period of culture, as compared to the cells applied by either LIPUS or G-PLGA alone. Our results suggest that the LIPUS stimulation could be used for osteogenic differentiation and, by the addition of a controlled-release particulate system of growth factors, it could give stable cell differentiation. In conclusion, the present investigation convincingly demonstrates that the ultrasound stimulation and growth factor supply system with polymeric vehicles can synergistically differentiate the stem cells and provide stable cell growth for the ex vivo therapy. Finally, the ultrasound stimulation with the controlled supply system of growth factors could be used as a noninvasive, efficient tool to provide a sufficient number of well-differentiated stem cells in the bone regeneration therapy.

## Figures and Tables

**Table 1:** Hydrodynamic size and zeta potential measurements of growth factor-containing PLGA types by the dynamic light scattering (DLS) method (*n* = 3).

Formulation		G-NP50/50	G-NP65/35	G-NP75/25
**Particle size (nm)**				
Solution in water	Day 1	498.4 (±15.2)	532.3 (±10.5)	562.7 (±12.4)
	Day 30	485.2 (±10.4)	510.2 (±4.4)	520.0 (±8.3)
Spray-dried powder	Day 1	502.1 (±8.4)	542.4 (±7.2)	534.6 (±9.5)
	Day 30	495.0 (±11.2)	504.2 (±6.4)	515.5 (±10.8)
**Zeta potential (mV)**				
Solution in water	Day 1	−8.2 (±2.5)	−5.2 (±3.2)	−8.2 (±3.2)
	Day 30	−9.7 (±1.8)	−7.1 (±2.8)	−7.5 (±2.1)
Spray-dried powder	Day 1	−7.5 (±3.5)	−6.3 (±1.4)	−8.6 (±2.7)
	Day 30	−8.5 (±2.4)	−8.2 (±2.2)	−9.5 (±1.5)

Standard deviation (SD) values are reported in the parenthesis for the averaged values.
